# The resident-as-teacher educational challenge: a needs assessment survey at the National Autonomous University of Mexico Faculty of Medicine

**DOI:** 10.1186/1472-6920-10-17

**Published:** 2010-02-16

**Authors:** Melchor Sánchez-Mendiola, Enrique L Graue-Wiechers, Leobardo C Ruiz-Pérez, Rocío García-Durán, Irene Durante-Montiel

**Affiliations:** 1 Secretaría de Educación Médica, Facultad de Medicina de la Universidad Nacional Autónoma de México, Ave. Universidad 3000, Ciudad Universitaria, México, D.F., 04510 México; 2 Dirección, Facultad de Medicina de la Universidad Nacional Autónoma de México, México, D.F., México; 3 División de Estudios de Posgrado e Investigación, Facultad de Medicina de la Universidad Nacional Autónoma de México, México, D.F., México; 4 Secretaría del Consejo Técnico, Facultad de Medicina de la Universidad Nacional Autónoma de México, México, D.F., México

## Abstract

**Background:**

The role of residents as educators is increasingly recognized, since it impacts residents, interns, medical students and other healthcare professionals. A widespread implementation of resident-as-teacher courses in developed countries' medical schools has occurred, with variable results. There is a dearth of information about this theme in developing countries. The National Autonomous University of Mexico (UNAM) Faculty of Medicine has more than 50% of the residency programs' physician population in Mexico. This report describes a needs assessment survey for a resident as teacher program at our institution.

**Methods:**

A cross-sectional descriptive survey was developed based on a review of the available literature and discussion by an expert multidisciplinary committee. The goal was to identify the residents' attitudes, academic needs and preferred educational strategies regarding resident-as-teacher activities throughout the residency. The survey was piloted and modified accordingly. The paper anonymous survey was sent to 7,685 residents, the total population of medical residents in UNAM programs in the country.

**Results:**

There was a 65.7% return rate (5,186 questionnaires), a broad and representative sample of the student population. The residents felt they had knowledge and were competent in medical education, but the majority felt a need to improve their knowledge and skills in this discipline. Most residents (92.5%) felt that their role as educators of medical students, interns and other residents was important/very important. They estimated that 45.5% of their learning came from other residents. Ninety percent stated that it was necessary to be trained in teaching skills. The themes identified to include in the educational intervention were mostly clinically oriented. The educational strategies in order of preference were interactive lectures with a professor, small groups with a moderator, material available in a website for self-learning, printed material for self-study and homework, and small group web-based learning.

**Conclusions:**

There is a large unmet need to implement educational interventions to improve residents' educational skills in postgraduate educational programs in developing countries. Most perceived needs of residents are practical and clinically oriented, and they prefer traditional educational strategies. Resident as teachers educational interventions need to be designed taking into account local needs and resources.

## Background

Residents play a critical role in real life medical education, it has been reported that they spend about 20% of their time teaching [1]. The importance of that role is not proportional to the amount of their training in educational processes, which is paradoxical since they are reported to be responsible for up to 80% of the education of interns and medical students [2]. Another relevant fact is that about two-thirds of residents receive more than 40% of their education from other residents [3]. Residents spend more time in direct contact with the health care institutions in-training personnel than many specialists or attending physicians, and they influence greatly the "hidden curriculum" of the educational activities in daily clinical care.

The postgraduate medical residency training period provides an excellent window of opportunity to target for educational skills improvement, as has been amply shown in the medical education literature [2,4]. There has been a growing recognition of the need to implement *"residents-as-teachers" *programs in graduate medical education, mainly in developed countries [5]. This trend has spawned several educational initiatives to improve residents' teaching skills, with a wide variety of curricula and teaching methodologies, producing variable results [6].

One of the critical factors for success in the planning and implementation of educational interventions is the assessment of the learners' needs, which for several reasons is not frequently done in postgraduate medical education [7]. Needs assessment is crucial to provide a foundation upon which a course or workshop can be built, and to assure that the educational intervention is relevant to the residents' needs and attainable in the local setting. There are some studies that describe residents' learning needs for teaching skills courses, but they are based in developed countries' educational institutions [8,9]. There is a paucity of published information about resident-as-educator initiatives in developing countries. As a first step in a strategy to develop effective educational interventions for residents' teaching skills, the Postgraduate Studies Division staff at the National Autonomous University of Mexico (UNAM) Faculty of Medicine designed a questionnaire to survey our postgraduate students' learning needs. The objective of the study was to explore the hypothesis that Mexican medical and surgical residents are conscious of the importance of their educational role in healthcare institutions and medical schools, and to identify their learning needs for a resident as educator program. This paper describes the results of the survey in a broad sample of UNAM residents in Mexico.

## Methods

### Study design

A cross-sectional non-experimental descriptive survey approach was used, with a self-completed paper questionnaire that was filled anonymously. The questionnaire explored opinions, attitudes and preferences about the residents' teaching role and training in medical education.

### Study setting

UNAM Faculty of Medicine is the largest medical school in Mexico and Latin America and is located in Mexico City. It is a public institution that serves socioeconomically diverse communities from all over the country, with a student population of more than 15 thousand, almost 8 thousand of which are specialty residents. The Postgraduate Studies Division of UNAM's Faculty of Medicine is the educational and administrative entity that coordinates the residency programs http://www.fmposgrado.unam.mx The Division has under its educational umbrella more than 50% of the residents in the country, distributed in more than 500 courses in 74 medical specialties and 93 training sites, that include public and private hospitals. The residents are distributed in all the Mexican states, although the majority are in Mexico City and surrounding areas.

### Questionnaire design and survey implementation

The first step was the creation of a formal Postgraduate Medical Education Committee, with explicit support from the medical school authorities. The committee included clinical opinion leaders, medical educators, and residency program directors. A review of the published research about the resident as educator concept was performed, and the relevant results were disseminated and discussed in the committee. After several sessions a needs assessment survey for residents was designed including some questions from the instruments described in the identified papers, and others that were deemed appropriate by the committee members. The draft was reviewed by the committee for content validity, and was piloted for clarity and feasibility in a group of 30 residents and modified accordingly.

The final questionnaire had three parts: part 1 included demographic data (age, gender, residency name, training site and academic year); part 2 had 14 questions about the perception of their teaching roles, the time dedicated to teaching, the relative educational importance of nurses, medical students, interns and other residents, the self-assessed teaching competence, an estimation of the learning that came from other residents and obstacles to the learning process in the hospital; part 3 asked the residents to rank the relative importance of 21 themes that could be included in a teaching skills educational intervention, questions about the preferred teaching methods for such a course and the time they would dedicate to it if it was online. All the questions were close-ended. The questionnaire is attached as an Appendix (Additional File 1: Resident as teacher needs assessment survey questionnaire).

The questionnaires were sent as a paper anonymous survey to the totality of our resident population in September 2005 (7,685 students). There was discussion about whether to use a random sampling methodology, but it was decided to send the instrument to all our resident population for symbolic purposes, so each and every postgraduate student at UNAM Faculty of Medicine was aware of the resident as educator concept and that the University authorities were planning on implementing such a program. Since we have 74 specialty courses in 93 training sites, if we had used a random sampling strategy many of the students, sites and/or courses would have had no notice about the survey and its implications. The packages were sent to the Director of Education of every UNAM residency program training site, with the number of questionnaires appropriate to the number of residents at each hospital. The documents included printed instructions for the survey administration. This scheme took advantage of the monthly administrative paperwork mailings which the Postgraduate Division at the University regularly sends all the training sites, so the strategy of sampling the totality of the resident population was relatively straightforward. Senior staff from the Postgraduate Studies Division personally contacted each hospital Director to emphasize the importance of the survey, and its potential educational implications for postgraduate medical education in Mexico, with specific instructions to administer the questionnaire in an anonymous manner and a collegial unthreatening atmosphere. The residents returned the questionnaire to the local Director of Education at their hospital, who was instructed to send back the filled questionnaires within a month after their receipt.

The anonymous completed questionnaires were sent back to the Postgraduate Division via the same administrative distribution mailings, and a written and telephone reminder was sent to the institutions that didn't return the questionnaires by the deadline date.

### Data analysis

The survey results data were analyzed with SPSS 15.0 for Windows (SPSS, Inc., Chicago, IL, USA). Data exploration and descriptive statistics were performed, and comparison among groups was done with inferential statistics. Comparison among categorical variables was done with chi-square and Kruskal-Wallis test, and among continuous variables with one-way ANOVA. For statistical tests a *p *value of less than 0.05 was considered statistically significant.

### Ethical considerations

Attempts were made to minimize students' non-participation, by offering an explanation of the study and the importance of their participation, without exerting undue influence. The instrument did not have individual student identifiers (names or matriculation numbers), to eliminate the risk of potential harm to the participants. In the event of their identities being recognized, all the information used in this study was harmless to the subjects. Ethical approval was obtained from the local Ethics committee, and care was taken to apply the World Medical Association Declaration of Helsinki principles of research http://www.wma.net/en/30publications/10policies/b3/17c.pdf.

## Results

### Demographics

A total of 5,186 questionnaires were returned, 133 were invalid (2.6%), leaving 5,053 documents to be included in the analysis. This represented a 65.7% return rate of the 7,685 sent questionnaires. The age of the respondents was 30.4 ± 7.8 years (mean ± SD). The gender distribution was 44.5% female and 55.5% male. These demographic data and the distribution of the residents in the 74 specialty courses and 93 training sites are similar to the data that the Postgraduate Studies Division has on file about the resident population at the time of the survey, providing evidence that the responders are a broad and representative sample of UNAM's postgraduate student population (Table 1).

**Table 1:** Demographic characteristics of survey respondents

**Age **in years (Mean ± SD)	30.4 ± 7.7			
		
Gender # (%)	Female 2,144 (44.5%)	Male 2,675 (55.5%)		
**Residency Year** **# of residents**	R1 = 1,638	R2 = 1,578	R3 = 1,152	>R3 = 685

Demographic characteristics of UNAM resident-as-teacher needs assessment survey respondents (n = 5,053). Numbers may not add exactly to total number of respondents in cases where residents didn't answer the question.

The most common specialty in the sample was family medicine (1,219 subjects, 24.1%), followed by pediatrics (406, 8.0%), internal medicine (327, 6.5%), obstetrics and gynecology (325, 6.4%), and 70 other specialty courses (Table 2).

**Table 2:** Specialty courses included in the survey

Specialty Course	Number of residents (%)
Family Medicine	1,219 (24.1)

Pediatrics	406 (8.0)

Internal Medicine	327 (6.5)

Gynecology and Obstetrics	325 (6.4)

Orthopedics	262 (5.2)

General Surgery	231 (4.6)

Anesthesiology	177 (3.5)

Cardiology	164 (3.2)

Psychiatry	144 (2.8)

Radiology	124 (2.4)

Ophthalmology	120 (2.4)

Rehabilitation Medicine	93 (1.8)

Otolaryngology	78 (1.5)

Dermatology	77 (1.5)

Critical Care Medicine	70 (1.4)

Allergy and clinical immunology	67 (1.3)

Neurosurgery	62 (1.2)

Neonatology	62 (1.2)

Urology	56 (1.1)

Plastic and reconstructive surgery	51 (1.0)

Oncologic surgery	49 (0.97)

Pediatric Surgery	44 (0.87)

Epidemiology	41 (0.81)

Pathology	40 (0.79)

Audiology and foniatrics	40 (0.79)

Gastroenterology	40 (0.79)

Infectology	33 (0.65)

Neurology	32 (0.63)

Neumology	32 (0.63)

Maternal-Fetal Medicine	31 (0.61)

Medical oncology	30 (0.59)

Pediatric cardiology	28 (0.55)

Hematology	28 (0.55)

Nephrology	27 (0.53)

Work medicine	26 (0.51)

Rheumatology	24 (0.47)

Cardiothoracic surgery	24 (0.47)

Angiology and vascular surgery	23 (0.45)

Medical genetics	22 (0.43)

Pediatric psychiatry	21 (0.41)

Reproductive medicine	19 (0.38)

Forensic medicine	17 (0.34)

Coloproctology	17 (0.34)

Endocrinology	16 (0.32)

Pediatric critical care medicine	15 (0.30)

Pediatric allergy and immunology	15 (0.30)

Nuclear medicine	14 (0.28)

Pediatric pulmonology	14 (0.28)

Pediatric neurology	14 (0.28)

Pediatric gastroenterology	13 (0.26)

Pediatric nephrology	13 (0.26)

Clinical pathology	12 (0.24)

Radio-oncology	12 (0.24)

Pediatric endocrinology	12 (0.24)

Sports medicine	12 (0.24)

Pediatric anesthesiology	11 (0.22)

Pediatric hematology	9 (0.18)

Pediatric oncology	9 (0.18)

Gynecologic urology	7 (0.14)

Neurologic endovascular therapy	6 (0.12)

Pediatric neurosurgery	5 (0.10)

Pediatric otolaryngology	5 (0.10)

Neuro-radiology	5 (0.10)

Clinical nutrition	4 (0.08)

Geriatrics	4 (0.08)

Pediatric pathology	4 (0.08)

Pediatric rheumatology	4 (0.08)

Pediatric dermatology	4 (0.08)

Neuro-otology	2 (0.04)

Neuropathology	2 (0.04)

Pediatric cardiothoracic surgery	2 (0.04)

Dermatopathology	2 (0.04)

Neuro-ophthalmology	2 (0.04)

Neuroanesthesiology	1 (0.02)

Number and percentage of residents in each specialty course that answered the survey questionnaire (n = 5,053).

The distribution of residents according to the year of training was a follows: 1638 first-year residents (R1 = 31.6%), 1578 second-year residents (R2 = 30.4%), 1152 third-year residents (R3 = 22.2%) and 685 above third-year residents (>R3 = 13.2%). This pattern reflects the distribution of the resident population in the Postgraduate Division files, suggesting that the sample is representative of the total population.

### Resident as educator: time, competence, roles and needs

**Figure 1:**
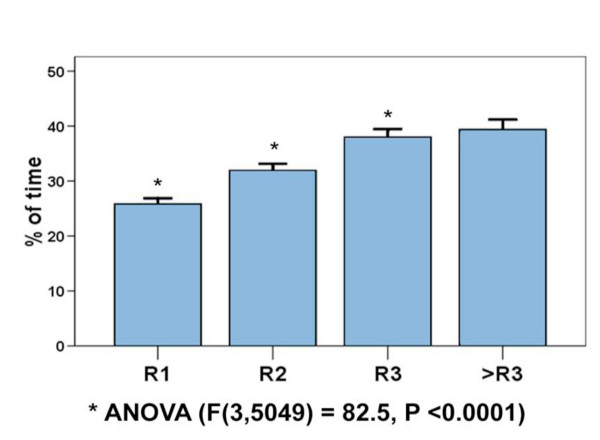
**Percentage of time dedicated to teaching by residency year**. Mean percentages of time dedicated to teaching and their 95% confidence intervals reported by the residents for each academic year.

When the residents were asked about the percentage of their time that they use educating other health care personnel (nurses, medical students, interns and other residents), the global mean was 32.3% (95% CI, 31.6 to 33.0). The mean percentages of time dedicated to teaching and its 95% confidence intervals for the different groups of residents according to their academic year are shown in Figure 1. There was a higher reported use of time teaching as they advanced in their program, from 25.8% in the first year to 38% in the third year. There was a significant difference among first, second and third year residents, with no difference in subsequent years in programs with more than three years duration (ANOVA with Student-Neuman-Keuls post-test comparison) [F(3,5049) = 82.5, p < 0.0001].

The residents estimated highly their knowledge of medical education, with a score of 7.06 (95% CI, 7.0 to 7.12) in a scale from 1 to 10 (1 = minimum, 10 = maximum). There was a small but statistically significant difference between men and women in this item, with males estimating their medical education knowledge 2.2% more than women [F(1,4817) = 13.5, p < 0.0001].

When estimating their competence to teach other residents the score was 6.36 (95% CI, 6.26 to 6.46). Sixty-two percent of the residents were in the 8 to 10 range (1 = not competent, 10 = very competent). Their self-assessed competence to teach other residents was significantly lower than the competence to teach interns, which was lower than the acknowledged competence to teach medical students and nurses (p < 0.001, Student-Newman-Keuls multiple comparisons test). There was no significant difference in the self-assessed competence to teach medical students and nurses. In all instances their mean declared competence was above six (Table 3).

**Table 3:** Self-assessed competence to teach different members of the healthcare team

	Mean	95% Confidence interval
**Residents**	6.36*	6.26 to 6.46

**Interns**	6.61**	6.52 to 6.71

**Medical students**	6.96***	6.87 to 7.05

**Nurses**	6.85	6.76 to 6.94

Student-Newman-Keuls multiple comparisons test:

*Residents vs. Interns, Medical students and Nurses p < 0.001

**Interns vs. Medical students and Nurses p < 0.01

***Medical students vs. Nurses p > 0.05

Descriptive statistics of the scores in self-assessed competence to teach residents, interns, medical students and nurses (1 = totally incompetent, 10 = totally competent), (n = 5,053).

Regarding the ranking of the time dedicated to teach other members of the health care team, the results are shown in Table 4. In this scheme, where 1 is more time and 4 is less time, their first priority were other residents, followed by interns, medical students and nurses, in that order (p < 0.001 for all comparisons, Kruskal Wallis with Dunn's multiple comparisons test).

**Table 4:** Ranking of time dedicated to teach different members of the healthcare team

	Mean	95% Confidence interval
**Residents**	1.42 *	1.38 to 1.45

**Interns**	1.74*	1.71 to 1.78

**Medical students**	2.05*	2.01 to 2.08

**Nurses**	2.47*	2.43 to 2.51

* Kruskal Wallis with Dunn's multiple comparisons test, p < 0.001 for all comparisons (residents, interns, students and nurses).

Ranking of time dedicated by residents to teach other residents, interns, medical students and nurses (1 = more time, 4 = less time), (n = 5,053).

When qualifying the statement *"residents have an important role in the education of other residents"*, 92% of them agreed or strongly agreed with this assertion. The results regarding their perception of their role as educators of the other members of the healthcare team are shown in Table 5 (where 1 = strongly agree, 5 = strongly disagree). The role of teaching other residents was deemed the most important, with a decreasing importance for interns, medical students and nurses, in that order.

**Table 5:** Perceptions of their role as educators of members of the healthcare team

	Mean	95% Confidence interval
**Residents**	1.31*	1.29 to 1.33

**Interns**	1.40**	1.38 to 1.42

**Medical Students**	1.55***	1.53 to 1.57

**Nurses**	2.14	2.11 to 2.17

Student-Newman-Keuls multiple comparisons test:

*Residents vs. Interns p > 0.05

*Residents vs. Medical students p < 0.01

*Residents vs. Nurses p < 0.001

**Interns vs. Medical students p > 0.05

**Interns vs. Nurses p < 0.001

***Medical students vs. Nurses p < 0.001

Perceptions of the importance of their role as educators of residents, interns, medical students and nurses (1 = strongly agree, 5 = strongly disagree), (n = 5,053).

When asked their opinion about the need for training in educational strategies during the residency program, almost 90% of the respondents agreed or strongly agreed with the need for this type of educational intervention, with an overall score of 1.49 (95% CI, 1.47 to 1.52) (1 = strongly agree, 5 = strongly disagree).

Ninety percent of them agreed or strongly agreed with the statement *"learning to teach better improves the quality of medical care" *with a score of 1.42 (95% CI, 1.40 to 1.44). They perceive the educational process by itself can impact clinical practice and potentially improve patient outcomes. Their opinion about the attitude of the medical specialists in the hospital to teaching was less enthusiastic, with a score of 2.11 (95% CI, 2.08 to 2.14). They also agree with the statement that the main obstacles to teaching about teaching in the residency are the lack of time and the excess of clinical work [1.95 (95% CI, 1.91 to 1.98)]. It is noteworthy that 16% of them disagree with this affirmation.

### The resident as teacher: content and methods

The section of the questionnaire that explored the importance of the potential themes to learn in a medical education program for residents asked them to rank 20 content areas in a scale of 0 to 4 (0 = the theme is not important, 4 = the theme is very important). The results are shown in Table 6. The most highly regarded subjects were how to teach psychomotor skills, diagnostic reasoning, the use of diagnostic tests and evidence-based medicine. They ranked highly how to give lectures and teaching during rounds. The subjects of motivation, learning theories and role modeling came out last, although it is important to point out that all the themes had a score above 2.9.

**Table 6:** Ranking of the importance of different themes to include in a resident as teacher educational intervention

Themes	Mean	95% Confidence interval
Teaching psychomotor skills	3.56	3.54 to 3.59

Diagnostic reasoning	3.55	3.52 to 3.57

Diagnostic tests	3.55	3.52 to 3.57

Evidence-based medicine	3.52	3.49 to 3.54

How to give lectures	3.40	3.37 to 3.43

Teaching during rounds	3.29	3.26 to 3.32

Feedback	3.24	3.22 to 3.27

Ethics	3.24	3.21 to 3.27

Communication skills	3.20	3.17 to 3.23

History & Physical	3.20	3.17 to 3.23

Time management	3.17	3.14 to 3.20

Burnout syndrome	3.14	3.11 to 3.17

Reflective practice	3.08	3.05 to 3.11

Learning styles	3.06	3.03 to 3.09

Bedside teaching	3.06	3.03 to 3.09

Leadership	3.05	3.02 to 3.08

Conflict management	3.03	3.00 to 3.06

Assessment methods	3.01	2.98 to 3.04

Motivation strategies	2.96	2.93 to 3.00

Learning theories	2.96	2.92 to 2.99

Role Modeling	2.90	2.87 to 2.93

List of the content ranked by residents as important to include in a resident as teacher educational intervention in a scale of 0 to 4 (0 = not important, 4 = very important).

When asked about their preferred teaching methods as learners of a "resident-as-teachers" educational intervention, the following results were obtained (Table 7): the most frequently requested method was interactive lectures with a professor, the second was small group sessions with a facilitator, followed by online individual learning. The less preferred methods were printed materials for self-directed study and online small groups. When asked the amount of time they would dedicate to the course if it was online, they answered 6.18 (95% CI, 5.95 to 6.40) hours per week. Regarding the percentage of their total learning experience in the residency that was due to residents' teaching, the response was 45.5 (95% CI, 44.8 to 46.2), with 26% of the residents estimating that their learning from other residents was above 70% of their total learning.

**Table 7:** Ranking of the preferred teaching methods for a resident as teacher educational intervention

Teaching Methods	Mean	95% Confidence interval
Interactive lectures with a professor	2.06	2.02 to 2.10

Small groups work with facilitator	2.20	2.16 to 2.23

Online individual learning	2.89	2.85 to 2.94

Printed material for self-directed study	2.97	2.93 to 3.01

Online small groups	3.54	3.49 to 3.58

Ranking of the preferred teaching methods to be used in a teaching skills course selected by our resident population (1 = most preferred, 5 = less preferred).

### The resident as teacher: medical vs. surgical specialties

The data were also explored by grouping the medical and surgical specialties, and comparing the responses of these two types of residents. The classification of surgical and medical courses was done by two of the authors, and was relatively straightforward with no disagreement. The results of this comparison were as follows:

• There was no difference in their declared knowledge of medical education or the percentage of time they used to teach.

• Their self-assessed competence to teach medical students [medical 6.82 (95% CI, 6.72 to 6.93); surgical 7.38 (95% CI, 7.19 to 7.57)] and residents [medical 6.27 (95% CI, 6.16 to 6.38); surgical 6.62 (95% CI, 6.43 to 6.81)] was higher for surgical residents, with no difference in the nurses and interns comparisons.

• Surgical residents ranked the time dedicated to teach other residents higher than medical residents [surgical 1.29 (95% CI, 1.22 to 1.36); medical 1.46 (95% CI, 1.41 to 1.50)], and medical residents ranked higher the time for interns, medical students and nurses than surgical specialties.

• Surgical residents had a higher perception of the importance of their role as educators of other residents than medical residents [surgical 1.19 (95% CI, 1.15 to 1.24); medical 1.35 (95% CI, 1.32 to 1.37)], and medical residents had a higher perception of their role as educators of nurses. There was no difference in the perception of their role as educators of medical students and interns.

• Medical residents rated the importance of training in teaching skills during residency higher than surgical residents [medical 1.44 (95% CI, 1.42 to 1.47); surgical 1.63 (95% CI, 1.58 to 1.67)].

• There were no statistical differences between surgical and medical specialties in the different themes to be included in a resident as teacher educational intervention.

• Medical residents declared that they would spend more hours per week in an online resident-as-teacher program than surgical residents [medical 6.4 (95% CI, 6.1 to 6.7); surgical 5.5 (95% CI, 5.2 to 5.8)].

• Surgical residents stated that they received a higher percentage of their total residency learning experience from other residents than their medical counterparts [surgical 50.4% (95% CI, 49.1 to 51.8); medical 43.6% (95% CI, 42.8 to 44.5)].

## Discussion

The results from this needs assessment survey at UNAM Faculty of Medicine in Mexico show that residents from many specialties dedicate a substantial amount of time to teaching activities, which increases as they progress through their academic programs. The residents are aware of the importance of their role as educators in the healthcare team, and feel a need for formal training in educational skills. The study explored the variety of educational content the residents would like to learn, found a preference for clinically oriented themes, and allowed ranking of their teaching modality preferences.

There is a broad body of research that describes the whole spectrum of the educational process of "resident-as-teachers" educational interventions, ranging from educational rationale, needs assessment, curriculum development, teaching methods, observational and experimental intervention studies, and reviews [2,4,10,11], from several developed countries including USA, Canada, Australia and others, but no studies have been published about this type of educational intervention in the setting of developing countries.

Residents are adult learners by nature and live a dual role as postgraduate students and healthcare workers, this situation creates a complex and challenging educational scenario. At the same time that they are full-time postgraduate students in a medical or surgical specialty sponsored by a university, they are healthcare workers that provide professional medical care employed by an academic health center. As a consequence of these multiple roles they participate in all the activities intrinsic to hospital life such as clinical care, research, education and many clerical and administrative functions. They fulfill all these functions struggling for balance between the academic role, the practical day-to-day clinical care role and their personal life. In this busy and complex environment, residents teach each other and all the members of the healthcare team, including nurses, medical students and other residents. There is an opportunity to improve the residents' teaching skills via educational interventions in the healthcare setting. Formal courses and workshops can improve the quality of the learning processes where the residents participate, as has been shown experimentally [4,12].

It is not unusual that learners' needs are not appropriately taken into account for planning educational programs, which is frequent in medical education postgraduate training in developing countries. In these settings most of the curriculum planning and design is done based on faculty expert opinion, following normative needs from external organizations and prescribed needs from the program planners. In the present era of learner-centered education it is imperative that the students' perceived and expressed needs are taken into account for quality education [7]. Felt or perceived learner needs are what the individuals or the group have identified as what they want to learn, and these needs are determined by their knowledge, attitudes, experience and where they work (what the students *think *they want to learn). Expressed needs are what a group or an individual articulate and express as their learning needs (what the students *say *they want to learn), which can be different from the perceived needs if there are some barriers for their expression [7]. The explicit needs assessment of the learners' needs is extremely useful for identifying educational goals and planning the educational intervention in an appropriate manner for the targeted learners, as is the case for medical and surgical residents.

One of the critical first steps for planning educational interventions is to perform a needs assessment, with a systematic collection and analysis of data that ensure the educational goals are aligned with the needs and are realistically achievable with the available resources. There are several approaches for performing a needs assessment, such as questionnaire surveys, focus groups, job or task analysis, performance review, and others [7]. We focused initially in a questionnaire survey, to obtain written responses to specific questions developed by a panel of experts.

This study has the largest sample size reported to date about the resident as educator theme, and is the first published work that explores this area of postgraduate education in developing countries [4,8,9]. Our population sample is the largest and most diverse in a needs assessment survey for residents' educational skills in the medical and educational literature, and reflects the demographic composition of the resident population at UNAM Faculty of Medicine. The 65.7% questionnaire return rate suggests the strategy for survey implementation was effective, since it is a high response rate for a mailed paper questionnaire sent to almost a hundred training sites all over the country. There is no universally accepted suitable response rate in surveys because that depends on many factors, but following the strategies for ensuring optimal response rate reported in the literature we obtained a reasonable rate: using the right kind of sponsors, appropriate inducement, the questionnaire format, the cover letter and a follow up strategy. Survey experts report minimum sample sizes for selected populations [13]. For example for populations of 5 to 10 thousand, appropriate samples of the whole population that have 95% confidence with 5% margin of error range from 357 to 370, so it can be argued that our sample of more than five thousand is an appropriate representation of the residency programs population in Mexico.

Unlike other published resident as educator needs assessment papers that included mostly one or a few specialties (surgery, family medicine, internal medicine and pediatrics) [8,9,11], our study explored 74 specialty and subspecialty courses in a variety of settings in Mexico (urban and community hospitals, public and private medical centers), adding information previously not reported in the literature for many medical and surgical specialties. The pattern of responses was slightly different in some of the questionnaire items for medical and surgical specialties, suggesting some differences in perception between these groups of residents, but the results were very similar overall, supporting the need for training in medical education throughout all the specialties.

The gender distribution is also in accordance with the global trend of increasing number of women in medicine [14]. At UNAM's Faculty of Medicine the female undergraduate population is more than 60% of the student body, and in the postgraduate setting the proportion is 44.5%, with an increasing trend in the last few years. The gender distribution is important in postgraduate medical education because there are many specialties that are more "feminized" than others, and there is evidence that gender can determine differences in social and cognitive aspects of medical education, which should be taken into account when designing the educational interventions [14].

The most common specialties found in this study reflect the distribution of residents in our programs, with the generalist residencies first (family medicine, internal medicine and pediatrics) composing 37% of the sample, followed by obstetrics and gynecology, orthopedics and general surgery. This distribution adds external validity to the findings, since they reflect the opinions of a wide variety of residents and is not limited to only a few specialties. Our sample includes residents from all academic years, since there is evidence that all residents participate in the educational process, although their teaching responsibilities are higher in the last years of their programs [2,11].

An important argument that bolsters the need for improving residents' teaching skills is the amount of time they dedicate to teaching, which in previous papers has been estimated from 20 to 25% [1,15]. In our study the mean reported time used in teaching was 32%, higher than previously estimated, and there was a higher reported use of time in teaching as they advanced in their residency programs. Our first year residents reported 25.8%, similar to published studies, but third year residents reported a significantly higher percentage of time, in average 38%, which is substantially higher than the published data. Hospitals in developing countries have a limited number of specialists in the staff, and their salaries are lower when compared to private practice fees. These conditions produce a scenario where clinical professors frequently need to work in private practice for a substantial amount of their time, leaving the residents in charge of the patients. This could partially explain the large amount of time they report using in the teaching process, which increases as they progress in the program.

Their estimated knowledge of medical education was rated well, as is usually the case when physicians are asked to rate their competence in an aspect of their training which they know is important. We found a small but statistically significant gender difference that reflects the different perceptions of competence (being higher in males).

Our survey found that residents estimated their competence to teach above the midpoint of the scale (more than 60% of them in the 8 to 10 range, where 10 is very competent), and that these self-reported competencies were lower for teaching residents than interns, which were also lower than their competence to teach medical students and nurses. The detected differences were quantitatively small, although statistically significant, probably due to the large sample size. Residents rated well their competence to teach since they are expected to be good teachers, and it's an activity they have performed and witnessed in other residents since the early stages of their training in medical school. The differences in their acknowledged competencies to teach the different members of the healthcare team are possibly related to the perception that teaching medical students and nurses is "easier" or requires less knowledge and teaching skills than teaching interns or residents, which are higher in the academic ladder.

Residents ranked other residents as their highest priority when it comes to teaching other members of the healthcare team, followed by interns, medical students and nurses. It is apparent that they dedicate more time to their direct peers, and less time to nurses and medical students, which is logical since in order to delegate functions in direct patient care and the performance of procedures they need to train their peers and the lower-year residents.

In agreement with the literature, we found that a high proportion of our residents think that they have an important role in the education of other residents, interns, medical students and nurses [8,9]. It is interesting that there is also a gradient of perceived importance in this regard, with other residents and interns being more important than nurses.

During the development of the questionnaire and the literature review, a consensus emerged that residents have a critical role in the education process in medical centers, and that it has been largely undervalued and unrecognized. It is important that their educational role be identified and that creative strategies are employed to improve its recognition, disseminating the resident-as-educator concept to all members of the healthcare team, as well as to society at large.

The majority of the residents in our sample think that there is a need for training in teaching during the residency program, which is in agreement with the published studies [8,9,11]. It is noteworthy that residents feel competent to teach while identifying a need for training in education. In our study residents think that training in education can improve clinical care and patient outcomes, thus making the link between the educational process and the quality of medical care. On the other hand, and also in agreement with the literature, they think that the attitude of professors to the residents' teaching role is not concordant with the importance of that role [8,9,11]. The fact that some of the main obstacles to teaching is the lack of time and intense clinical work underscore the idea that educational interventions need to be tailored to complex scenarios with multiple time demands, and integrated into the clinical work flow for appropriate context.

There are several papers that discuss the content of the curricular programs for resident as teacher educational interventions, and our results are in agreement with most of these findings [2,11,12,16,17]. There are many themes of potential interest to residents and faculty that could be covered in a resident as educator curriculum, but sometimes in education "less is more", we should try to cover the essential topics in an educationally efficient manner. Our findings suggest that we should include practical and clinically oriented themes in a resident as educator intervention, and decide what content to cover using our results plus the unperceived learners' needs identified by the faculty and the Postgraduate Studies Committee experts. In doing so, we should strive to balance the perceived and expressed needs with the normative and prescribed needs. The ranking of the contents identified in our survey will be helpful to design the educational intervention in the local setting.

A crucial aspect of the planning and implementation of a teaching skills improvement program for residents is the selection of the teaching modality, which needs to balance the preferences of the students with the available resources [18,19]. Our findings are similar to other authors [8], with a preference for interactive conferences with experts and small group sessions with facilitators. The residents in our sample do not seem to prefer online sessions.

The percentage of the total learning experience during residency that came from other residents was estimated at 45.5%, a number similar to those previously reported [2,3], and it is noteworthy that a quarter of our residents estimated this number to be above 70%. This is suggestive of evidence that residents taught them a great portion of their specialty acquired knowledge, attitudes and skills.

Our study has several strengths: it has a large sample size, and represents a broad and diverse sample of the graduate medical student population in Mexico, including residents from multiple specialties; the response rate to the questionnaire was satisfactory for a study of this nature and scope; it used an instrument developed in an iterative process with a committee of experts in clinical medicine and postgraduate medical education, providing evidence of construct validity; and it was anonymous, decreasing the potential for bias in the residents' responses.

Some of the limitations of the study and its potential effects are: the sampling included only residents from UNAM Faculty of Medicine postgraduate programs, and although UNAM residents come from different private and public medical schools, students from other universities in Mexico could have a different opinion of the importance of their educational role and their needs; we used as the only modality of learning needs assessment a written questionnaire, there is a need to triangulate with other strategies like structured interviews or focus groups, which could identify other themes; the study has the limitations inherent to the survey questionnaire method, it elicits responses to specific questions, and it's difficult to be sure if the respondents understood clearly what was being asked, the information is limited to what was asked and how, this could leave some relevant areas unexplored; another limitation related to questionnaires is that they provide self-assessed needs and estimates, which can partially echo their real needs, and can be difficult to generalize since they reflect the answers and views of only the responders, which could overestimate their teaching skills [13,20]. It is uncertain if the non-responders had different ideas or opinions, the survey questionnaire was anonymous so it would have been logistically difficult to sample the non-responder population in our residency programs settings.

We intend to follow this investigation with the design and implementation of a resident-as-teacher workshop, based on our data and the educational interventions already available in other countries, adapting it to our local needs and resources. The workshop will be piloted in one of our residency sites, in order to be implemented in cascade in other hospitals. We also plan to develop a resident-as-teacher website, with material in Spanish to be used as an educational supplement and resource center for our professors and residents.

After a thorough search of several medical and educational databases, with no language restrictions and including Latin America and Mexican sources, we didn't find any published paper about the resident as teacher concept, so we posit that the data we present are the first to analyze this issue in developing countries. For the reasons described previously, we believe that the residents' manpower in developing countries healthcare institutions is a vastly underused educational force. It is important to explore this issue in other developing countries, and to plan, design and implement educational interventions to improve our residents' teaching skills.

## Conclusions

Medical residents in Mexico dedicate a substantial amount of their time to teaching, as reported in other countries, and this effort increases as they progress through the residency program. The residents are aware of the importance of their role as educators of nurses, medical students, interns and other residents, and they have a need for formal training in their educational skills.

They want to learn several subjects related to medical education, and there is a preference for those that are more clinically oriented. They prefer traditional lectures with an expert as the teaching methodology for a resident as teacher educational intervention, and they receive almost half of their learning from other residents.

Resident as teachers educational interventions need to be designed taking into account local needs and resources. The effective use of this educational opportunity to improve healthcare quality and medical education in hospital settings should be one of the priorities of medical schools and academic health centers.

## Competing interests

The authors declare that they have no competing interests.

## Authors' contributions

MSM, EGW, LCR and IDM were responsible for the study conception and design, as well as the design of the questionnaire. MSM, LCR and RCG participated in the data collection and quality control processes. MSM and RCG conducted the data analysis and drafted the manuscript. All authors made critical revisions of the paper, read and approved the final manuscript.

## Pre-publication history

The pre-publication history for this paper can be accessed here:

http://www.biomedcentral.com/1472-6920/10/17/prepub

## Supplementary Material

Additional file 1 **Resident as teacher needs assessment survey questionnaire**. Survey questionnaire designed by the Postgraduate Medical Education Committee, UNAM Faculty of Medicine, attached as a Microsoft Word document. Click here for file
