# Supplementary material for: The resident-as-teacher educational challenge: a needs assessment survey at the National Autonomous University of Mexico Faculty of Medicine
Source: BMC Med Educ. 2010 Feb 16;10:17. doi: 10.1186/1472-6920-10-17 (PMC2830225; doi:10.1186/1472-6920-10-17)
Supplement: Additional file 1 — Resident as teacher needs assessment survey questionnaire. Survey questionnaire designed by the Postgraduate Medical Education Committee, UNAM Faculty of Medicine, attached as a Microsoft Word document. [file 1472-6920-10-17-S1.DOC]

| **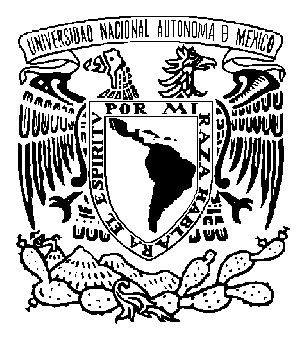** | **NATIONAL AUTONOMOUS UNIVERSITY OF MEXICO**  **FACULTY OF MEDICINE**  **POSTGRADUATE STUDIES DIVISION**  **“RESIDENT AS TEACHER” QUESTIONNAIRE** | **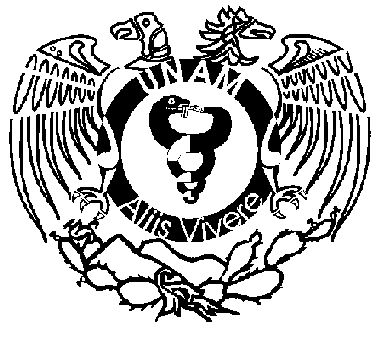** |
| --- | --- | --- |

# DATE: ______________

| RESIDENCY SITE: |  | AGE: |  | YEARS |
| --- | --- | --- | --- | --- |

GENDER: 1. Female: 2. Male:

| RESIDENCY SPECIALTY: |  |
| --- | --- |

*Mark with “X” the year of residency program you are currently taking:*

R1 ______ R2 ______ R3 _____ >R3 _____

1. How much do you know about the field of medical education? (in a scale of 1 to 10, where

1 = no knowledge, 10 = maximum knowledge)

2. What percentage of time during residency do you use teaching members of the healthcare

team? (medical students, nurses, interns, other residents)

%

3. Rank from 1 to 4 the healthcare personnel whom you teach, where 1 is the group you use more

time teaching and 4 less time teaching:

A) Nurses ______

B) Medical Students ______

C) Interns ______

D) Other Residents ______

4. How would you rate your competency to teach the following members of the healthcare team (in

a scale of 1 to 10, where 1 = totally incompetent and 10 = totally competent):

A) Nurses ______

B) Medical Students ______

C) Interns ______

D) Other Residents ______

For items 6 to 14, choose the option that better reflects your opinion about the role of the resident as a teacher:

1) Strongly agree

2) Agree

3) Neither agree or disagree

4) Disagree

5) Strongly disagree

| 6. | Residents have an important role teaching nurses |  |  |
| --- | --- | --- | --- |
|  |  |  |  |
| 7. | Residents have an important role teaching medical students |  |  |
|  |  |  |  |
| 8. | Residents have an important role teaching interns |  |  |
|  |  |  |  |
| 9. | Residents have an important role teaching other residents |  |  |
|  |  |  |  |
| 10. | It is important to be trained in teaching skills during residency |  |  |
|  |  |  |  |
| 11. | Learning how to teach better can improve the quality of medical care |  |  |
|  |  |  |  |
| 12. | Attending physicians and residents have a good attitude to teaching in your hospital |  |  |
|  |  |  |  |
| 13. | The main obstacle to having teaching skills sessions during residency programs is the lack of time |  |  |
|  |  |  |  |
| 14. | The main obstacle to having teaching skills sessions during residency programs is the excess of work |  |  |

If a teaching skills workshop was implemented in your residency program, which of the following topics would you like to see included? (Rate the topics from 0 to 4, where 1 is not important and 4 is very important, write 0 if you don't know about the topic)

| 15. |  | Motivation strategies |
| --- | --- | --- |
|  |  |  |
| 16. |  | Leadership |
|  |  |  |
| 17. |  | Learning theories |
|  |  |  |
| 18. |  | Learning styles |
|  |  |  |
| 19. |  | Communication skills |
|  |  |  |
| 20. |  | How to give lectures |
|  |  |  |
| 21. |  | Feedback |
|  |  |  |
| 22. |  | Role model |
|  |  |  |
| 23. |  | Burnout syndrome |
|  |  |  |
| 24. |  | Assessment methods |
|  |  |  |
| 25. |  | Conflict management |
|  |  |  |
| 26. |  | Bedside teaching |
|  |  |  |
| 27. |  | History & Physical |
|  |  |  |
| 28. |  | Diagnostic reasoning |
|  |  |  |
| 29. |  | Diagnostic Tests |
|  |  |  |
| 30. |  | Teaching psychomotor skills |
|  |  |  |
| 31. |  | Evidence-based medicine |
|  |  |  |
| 32. |  | Reflective practice |
|  |  |  |
| 33. |  | Teaching during rounds |
|  |  |  |
| 34. |  | Time management |
|  |  |  |
| 35. |  | Ethics |
|  |  |  |
| 36. |  | Other(s), especify: ____________________________________________________ |

1. Rate in ascending order (1 for the preferred method, 5 for the least preferred) the teaching methodologies you would prefer to use in a “Resident as teacher” educational activity for residents:

_____ Interactive conferences with a teacher

_____ Work in small groups with a facilitator

_____ Printed readings for self study and programmed assignments

_____ Didactic material available in a Web site for online individual learning

_____ Online work in small groups

1. If the “Resident as teacher” educational activity were online, how many hours per week would you dedicate to it?

Hours

1. Estimate your total learning experience during the residency years (knowledge, attitudes and skills), what percentage of that learning is due to your fellow residents?

%
